# Reply to: First Brazilian recommendation on physiotherapy with sensory motor stimulation in newborns and infants in the intensive care unit

**DOI:** 10.5935/0103-507X.20220033-en

**Published:** 2022

**Authors:** Cíntia Johnston, Mônica Carvalho Sanchez Stopiglia, Simone Nascimento Santos Ribeiro, Cristiane Sousa Nascimento Baez, Silvana Alves Pereira

**Affiliations:** 1 Faculdade de Medicina, Universidade de São Paulo - São Paulo (SP), Brazil.; 2 Hospital da Mulher Prof. Dr. José Aristodemo Pinotti, Universidade Estadual de Campinas - São Paulo (SP), Brazil.; 3 Faculdade de Ciências Médicas de Minas Gerais - Belo Horizonte (MG), Brazil.; 4 Instituto Federal de Educação, Ciência e Tecnologia do Rio de Janeiro - Rio de Janeiro (RJ), Brazil.; 6 Universidade Federal do Rio Grande do Norte - Natal (RN), Brazil.

**TO THE EDITOR**

Historically, the roles and skills required for neonatal physical therapists were developed by the Pediatric Section of the American Physical Therapy Association (APTA) and were first published in 1989.^(1)^ They were expanded in a 1999 publication ^(2)^ and updated in two publications by Sweeney et al.,^(3,4)^ in the same Pediatric Section of APTA, reviewing functions, competencies, theoretical structures, emerging literature database and recommendations of evidencebased practices of neonatal physical therapy. These updates reflect the needs of contemporary neonatal physical therapy practice, respected by the authors in the preparation of the First Brazilian Recommendation of Physiotherapy for Sensory-Motor Stimulation for Newborns and Infants in Neonatal Intensive Care Units. ^(5)^

To work in the neonatal intensive care unit (ICU), the physiotherapist needs specific training and refined skills in the evaluation, interpretation and modification of his or her conduct or continuous resequencing of physical therapy procedures aimed at infants with structural, physiological and behavioral vulnerabilities, which predispose them to instability during routine procedures.^(3)^ The physical therapy approach should include evidence-based interventions and focus on care for the baby and his or her family.^(3,4)^

Other relevant international publications also support the evidence-based practice of physical therapy in the neonatal ICU to provide adequate care for developing infants and families in the neonatal ICU on a continuous basis.^(6-11)^

It is understood that the experts who participated in the development of the document have unquestionable technical and scientific capacity to prepare any document based on scientific evidence in the field of neonatal intensive care. In addition to the questions, the First Brazilian Physical Therapy Recommendation for Sensory-Motor Stimulation for Newborns and Infants in a Neonatal Intensive Care Unit^(5)^ aims to describe the methods of sensorimotor stimulation and their levels of scientific evidence, and suction is a positive finding described in some of the included studies. The document did not aim to propose or teach physical therapy protocols or any other professional area, and there was no intention to simplify or maximize any intervention or finding, in addition to what was found in the scientific studies included in the recommendation (see inclusion criteria for the study).

All authors work in collaboration with speech therapists in routine care in the neonatal ICU and reinforce the esteem of the professional area and colleagues.
